# Hybrid FSK-PSK Waveform Optimization for Radar Based on Alternating Direction Method of Multiplier (ADMM)

**DOI:** 10.3390/s21237915

**Published:** 2021-11-27

**Authors:** Zhiting Fei, Jiachen Zhao, Zhe Geng, Xiaohua Zhu, Jindong Zhang

**Affiliations:** 1 School of Electronic and Optical Engineering, Nanjing University of Science and Technology, Nanjing 210094, China; zxh@njust.edu.cn; 2 Research and Development Department, China Academy of Launch Vehicle Technology, Beijing 100076, China; 3 College of Electronic Information Engineering, Nanjing University of Aeronautics and Astronautics, Nanjing 210016, China; felixwin@nuaa.edu.cn (J.Z.); zhegeng@nuaa.edu.cn (Z.G.); zhangjd@nuaa.edu.cn (J.Z.)

**Keywords:** hybrid modulated signal, waveform optimization, alternating direction method of multiplier, intra-pulse slice repeater jamming

## Abstract

In this paper, a new radar signal modulated with a hybrid of the frequency shift keying (FSK) and the phase shift keying (PSK) signal—i.e., the FSK-PSK signal—is studied. Different phase encoding sequences are used to modulate the sub-pulses to obtain lower sidelobe levels and ensure signal orthogonality. In addition, to counter intra-pulse slice repeater jamming of specific length generated by the enemy jammer, an orthogonal waveform made of sub-pulses of equal length based on the FSK-PSK modulation scheme is designed. The simulation results show that the optimized discrete phase encoding sequence can significantly enhance the orthogonality of the sub-pulse in the FSK-PSK signal and effectively suppress the slice repeater jamming. Two algorithms are proposed: (1) the low sidelobe waveform optimization algorithm based on ADMM (LSW-ADMM); and (2) the anti-slice-repeater-jamming algorithm based on ADMM (ASRJ-ADMM). Both algorithms exhibit fast convergence speed and low computational complexity.

## 1. Introduction

In modern radar systems, the characteristics of radar waveform contributes greatly to various radar performance metrics, such as the detection probability, the resolution, the measurement accuracy, the probability of intercept, and anti-jamming capabilities. By optimizing the waveforms transmitted by radar, the radar detection and anti-jamming performance could be enhanced dramatically [1]. For example, the phased coded sequences set could be optimized for high peak-to-sidelobe level (PSL) and good quadrature performance [2,3].

Both phase shift keying (PSK) and frequency shift keying (FSK) are commonly used radar modulation schemes. Since the Doppler tolerance of the PSK signal is relatively low, it is not an optimum option for detecting target with high moving speed [4,5]. Compared with the PSK signal, the pulse compression ratio of the FSK signal is relatively high, which yields lower sidelobe levels. The hybrid modulation of FSK and PSK could be used to form a new class of modulated signal—i.e., FSK-PSK signal—which exhibits large time-bandwidth product and outstanding low probability-of-intercept (LPI) performance.

In recent years, extensive research has been carried out in the radar transmit waveform design area. In [6,7,8,9,10,11,12,13,14], in order to solve the problem of target masking caused by high sidelobes in the range-Doppler domain, different optimization algorithms have been designed for sidelobe suppression. Tao Fan [15] et al. designed a Doppler filter bank for non-uniform pulse repetition interval (PRI) radar to suppress clutters. For radar to coexist with other wireless systems, Khaled Alhujaili et al. optimized the transmit beam pattern for multiple-input multiple-output (MIMO) radar in [16]. Furthermore, Ehsan Raei et al. proposed a method for the joint design of the phase coding sequence and the Doppler filter at the receiver [17]. To minimize the side lobe level of the ambiguity function, the periodic phase sequence set is optimized in [18,19,20]. In [21], X. Wei et al. first established the slicing jamming model, then calculated the correlation coefficients to choose the appropriate location of the sampling window for jamming sampling, finally suppressed the slicing jamming with adaptive sidelobe cancellation. C. Zhou et al. designed a specific low-sidelobe phase-coded signal as the master signal, which was split into a number of sub-signals according to the modulation parameters of the repeater jamming. The mismatched filter was subsequently designed for the sub-signals via convex optimization to ensure the orthogonality between the different sub-signals [22]. In [23,24], an anti-jamming method was proposed, which was realized by generating the phase-coded (PC) signal and LFM-PC signal with high Doppler tolerance to improve the SAR imaging performance. S. Zhong [25] et al. designed an intra-pulse quadrature phase-encoded signal, and proposed a genetic algorithm based on the DNA coding to achieve low autocorrelation sidelobes and cross-correlations. In [26], S. Baher Safa Hanbali proposed an improved method based on both interrupted sampling repeater jamming (ISRJ) and frequency shifting modulation to protect the true target more effectively in the presence of amplitude mismatch between cancellation signal and true target echo. In [27], K. Zhou et al. proposed a method to suppress the ISRJ by jointly designing the radar waveform and mismatch filter.

The hybrid FSK-PSK modulated signal, which combines the advantages of the FSK signal and the PSK signal, exhibits good resolution, measurement accuracy, and anti-jamming performance. In recent years, it has been researched extensively in literature. In [28], Skinner et al. demonstrated the superiority of the Gaussian FSK signal modulated with pseudo-noise bipolar PSK sequence in the aspect of the range sidelobes. In [29], Donohoe et al. compared the ambiguity functions of various FSK signals, PSK signals and FSK-PSK signals modulated according to different coding schemes. The sidelobes of the ambiguity function associated with FSK-PSK signals are lower than those associated with the other two types of signals. In [30], each sub-pulse of the FSK-PSK signal is modulated with a pair of complementary Welti codes, while each sub-pulse performs frequency hopping according to the Costas array sequence. Compared with the Costas-coded FSK signal and the Welti-coded PSK signal, the FSK-PSK signal demonstrates lower ambiguity function sidelobes. In [31,32], Wong and Chung studied the characteristics of the FSK-PSK signal in many aspects—e.g., ambiguity function—and proposed a genetic algorithm (GA) to optimize the FSK-PSK signal. The simulation results show that the optimized FSK-PSK signal has lower autocorrelation function (AF) sidelobe levels and cross-correlations. In [33], J. Zhang et al. proposed a novel jamming detection and suppression method by exploiting the sub-pulse compression with hybrid FSK-PSK signals. Actually, limitations and conditions of the FSK-PSK signal exists in the aspects of ELINT (Electronic Intelligence) systems. [34] presents the procedure of radar source identification based on recognition of residually distinctive primal features using linear regression method (LRM) and concerns this problem.

The conventional FSK-PSK signals are usually modulated with intra-sub-pulse phase-coding and inter-sub-pulse frequency-coding—i.e., identical phase coding sequences are used for distinctive sub-pulses. To achieve low sidelobes and near-orthogonality, we propose a new FSK-PSK signal, which uses different phase-coding sequences to modulate each sub-pulse. To reduce the AF sidelobes while enhancing the anti-jamming capability of radar, two optimization problems are considered in this paper. The first is to optimize the autocorrelation function of the FSK-PSK signal so that low sidelobe levels are obtained. The second is to optimize the internal discrete phase encoding sequences of the sub-pulses so that the ultimate goal of anti-slice-repeater-jamming is realized.

To obtain an improved version of the FSK-PSK signal for enhanced detection performance and anti-jamming capability, this paper exploits the alternating direction method of multiplier (ADMM) algorithm [35]. The contribution of this work is summarized as follows:

(1) The low sidelobe waveform (LSW) optimization algorithm based on ADMM, i.e., LSW-ADMM, is proposed. Distinctive phase encoding sequences are used for different sub-pulses, which are optimized with the LSW-ADMM algorithm for sidelobe reduction. Compared with the conventional FSK-PSK signals modulated by the classical code sequences, a higher-order of randomness is achieved, which guarantees enhanced anti-jamming capability of the radar system.
(2) To encounter the intra-pulse slice repeater jamming, the ASRJ-ADMM algorithm is proposed. Each sub-pulse of the FSK-PSK signal, which is coded with a distinctive set of phases, performs frequency hopping according to the optimized sequence. By ensuring the orthogonality of the sub-pulses of the FSK-PSK signal, the intra-pulse slice repeater jamming is effectively suppressed.

Both optimization algorithms demonstrate fast convergence speed and low computational load. In numerical simulations, it is proved that the proposed algorithms outperform the widely adopted heuristic search algorithm, such as the GA algorithm.

The rest of this work is organized as follows. Section 2 presents the mathematical model of FSK-PSK signal, the AF of which is derived analytically. Section 3 proposes LSW-ADMM algorithm. Section 4 proposes the ASRJ-ADMM algorithm for countering intra-pulse slice repeater jamming. Section 5 verifies the outstanding performance of the proposed algorithm with extensive numerical simulations. Finally, concluding remarks and directions for future research are offered in Section 6.

## 2. FSK-PSK Hybrid Modulated Signal Model

The conventional FSK-PSK signal is modulated by intra-sub-pulse phase-coding and inter-sub-pulse frequency agility. A wide pulse is divided into several phase-coded sub-pulses that perform frequency-hopping according to a specific frequency-coding sequence.

### 2.1. Ambiguity Function of the FSK-PSK Hybrid Modulated Signal

**Figure 1:**
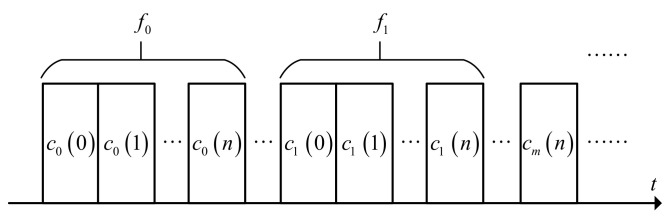
Schematic diagram of the proposed FSK-PSK signal.

To improve the waveform properties of the FSK-PSK signal and to ensure the near-orthogonality between the sub-pulses, we propose to use distinctive phase encoding sequences to modulate each sub-pulse. Figure 1 shows the partial structure of the FSK-PSK signal.

In Figure 1, $f_{m}$ represents the frequency hopping code of the *m*-th sub-pulse. If the length of the phase-coding sequence of the sub-pulse is N, then the phase-coding sequence for the *m*-th sub-pulse is $c_{m}^{}\left(n\right)=e_{}^{j2\pi k_{m}^{}\left(n\right)/K},n=0,1,\cdots ,N-1$, where K represents the number of discrete phases.

Suppose that the total pulse width of the FSK-PSK hybrid modulated signal composed of M sub-pulses is T, it follows that the sub-pulse width is $T_{c}=T/M$. Hence, the FSK-PSK hybrid modulated signal can be expressed as

(1) $$\begin{array}{cc} u_{\mathrm{FSK}-\mathrm{PSK}}^{}\left(t\right)=\sum_{m=0}^{M-1}v_{m}\left(t-mT_{c}\right), & 0\leq t\leq T \end{array}$$

where the *m*-th sub-pulse signal $v_{m}^{}\left(t\right)$ can be written as

(2) $$v_{m}\left(t\right)=e_{}^{j2\pi f_{m}^{}t}\sum_{n=0}^{N-1}c_{m}\left(n\right)p_{n}^{}\left(t\right)$$

In (2), $f_{m}=\sigma_{m}^{}/T_{P}^{},m=0,1,\cdots ,M-1$ is the frequency hopping code sequence, $\sigma_{m}^{}\in N_{+}^{}$ is the frequency hopping coefficient, $c={\left[c_{0}{}^{T},c_{1}{}^{T},\cdots c_{M-1}{}^{T}\right]}^{T}$ represents the coding sequence of *M* sub-pulses, and $p_{n}^{}\left(t\right)$ is the ideal rectangular pulse with a width of $T_{P}=T_{c}^{}/N$. The spectrum of the phase-coded signal is obtained as

(3) $$V_{m}^{}\left(f\right)=\sin c\left[\left(f-f_{m}^{}\right)T_{P}^{}\right]e^{-j\pi \left(f-f_{m}^{}\right)T_{P}}\left[\sum_{n=0}^{N-1}c\left(n\right)e^{-j2n\pi fT_{P}^{}}\right]$$

and the spectrum of the FSK-PSK signal is given by

(4) $$U_{\mathrm{FSK}-\mathrm{PSK}}^{}\left(f\right)=\sum_{m=0}^{M-1}V_{m}^{}\left(f\right)e_{}^{-j2m\pi fT_{c}^{}}$$

The FSK-PSK signal bandwidth $B_{\mathrm{FSK}-\mathrm{PSK}}^{}$ is approximately equal to

(5) $$B_{\mathrm{FSK}-\mathrm{PSK}}^{}\approx \left|f_{H}^{}-f_{L}\right|+\frac{1}{T_{P}^{}}$$

If the frequency hopping coefficients $\sigma_{m}$, m=0,1,⋯,M−1, are a series of continuous positive integers, then we have

(6) $$B_{\mathrm{FSK}-\mathrm{PSK}}^{}=\frac{N}{T_{P}^{}}=N\Delta f$$

The ambiguity function of the FSK-PSK signal can be obtained as:

(7) $$\chi_{\mathrm{FSK}-\mathrm{PSK}}\left(\tau ,\zeta \right)=\sum_{m=0}^{M-1}\sum_{l=0}^{M-1}\chi_{m,l}\left(\tau ,\zeta \right)$$

where m and l represent the sub-pulses corresponding to the *m*-th and the *l*-th frequency point, and $\left(l-m-1\right)T_{c}\leq \tau \leq \left(l-m+1\right)T_{c}$. $\chi_{m,l}\left(\tau ,\zeta \right)$ in (7) could be further written as:

(8) $$\begin{array}{c} \chi_{m,l}\left(\tau ,\zeta \right)\\ =\int_{-\infty }^{\infty }v_{m}\left(t-mT_{c}\right)v_{l}^{*}\left(t+\tau -lT_{c}\right)\cdot e_{}^{j2\pi \zeta t}dt\\ =\int_{-\infty }^{\infty }e_{}^{j2\pi f_{m}^{}t}\sum_{n=0}^{N-1}c_{m}\left(n\right)p_{n}^{}\left(t-mT_{c}\right)e_{}^{-j2\pi f_{l}^{}\left(t+\tau \right)}\sum_{r=0}^{N-1}c_{l}^{*}\left(k\right)p_{r}^{}\left(t+\tau -lT_{c}\right)e_{}^{j2\pi \zeta t}dt\\ =\sum_{n=0}^{N-1}\sum_{r=0}^{N-1}c_{m}\left(n\right)c_{l}^{*}\left(r\right)e_{}^{-j2\pi f_{l}^{}\tau }\chi_{m,n,l,r}\left(\tau ,\zeta \right) \end{array}$$

where n and r represent the *n*-th and the *r*-th code, and $\chi_{m,n,l,r}\left(\tau ,\zeta \right)$ is given by,

(9) $$\chi_{m,n,l,r}\left(\tau ,\zeta \right)=\int_{-\infty }^{\infty }p_{n}^{}\left(t-mT_{c}^{}\right)p_{r}^{}\left(t+\tau -lT_{c}^{}\right)e^{j2\pi \left(f_{m}-f_{l}+\zeta \right)t}dt$$

Given that $\left|\tau +\left(n-r\right)T_{P}+\left(m-l\right)T_{c}\right|\leq T_{P}$, (9) can be further simplified as

(10) $$\begin{array}{c} \chi_{m,n,l,r}\left(\tau ,\zeta \right)\\ =\left[T_{P}-\left|\tau +\left(n-r\right)T_{P}+\left(m-l\right)T_{c}\right|\right]e_{}^{j\pi \left(f_{m}-f_{l}+\zeta \right)\left[\left(n+r+1\right)T_{P}+\left(m+l\right)T_{c}-\tau \right]}\\ \times \mathrm{sinc}\left\{\left(f_{m}-f_{l}+\zeta \right)\left[T_{P}-\left|\tau +\left(n-r\right)T_{P}+\left(m-l\right)T_{c}\right|\right]\right\} \end{array}$$

The amplitude of the main peak of the FSK-PSK signal in the Doppler dimension is

(11) $$\left|\chi_{\mathrm{FSK}-\mathrm{PSK}}^{}\left(0,\zeta \right)\right|=\left|\sin c\left(\zeta T\right)\right|$$

Therefore, the Doppler tolerance of the FSK-PSK hybrid modulated signal is the same as that of FSK signal and PSK signal, which is expressed as

(12) $${\mathrm{DT}}_{\mathrm{FSK}-\mathrm{PSK}}=\frac{0.443}{T}$$

### 2.2. Autocorrelation Function of FSK-PSK Hybrid Modulated Signal

For the convenience of derivation, the FSK-PSK signal in (1)–(2) is rewritten as

(13) $$u\left(t\right)=\sum_{n=0}^{NM-1}c\left(n\right)p_{n}\left(t\right)e_{}^{j2\pi f_{n}^{}t}$$

where $f_{n}^{}=\sigma_{n}^{}/T_{P}^{},n=0,1,\cdots ,NM-1$ represents the *n*-th hopping frequency point and is expressed as

(14) $$f_{n}\in f={\left[\underset{N}{\underset{︸}{f_{0},f_{0},\cdots ,f_{0}}},\underset{N}{\underset{︸}{f_{1},f_{1},\cdots ,f_{1}}},\cdots ,\underset{N}{\underset{︸}{f_{M-1},f_{M-1},\cdots ,f_{M-1}}}\right]}_{}^{T}$$

$c\left(n\right)=e_{}^{j2\pi k\left(n\right)/K},n=0,1,\cdots ,NM-1$ in (13) represents the *n*-th phase code. The phase-coding sequence could be expressed as vector $c=[c\left(0\right),c\left(1\right),\ldots ,c\left(NM-1\right)]$.

Suppose that $N_{S}^{}$ points are sampled from *u*(*t*) in (13) with sampling period $T_{S}^{}=T_{P}^{}/N_{S}^{}$, and the total number of sampling points in a pulse is $N^{\prime }=NM\cdot N_{S}^{}$. Equation (13) could be rewritten as

(15) $$u\left(t\right)=\sum_{n=0}^{N^{\prime }-1}c^{\prime }\left(n\right){p^{\prime }}_{n}\left(t\right)e_{}^{j2\pi {f^{\prime }}_{n}^{}t}$$

where ${f^{\prime }}_{n}^{}={\sigma^{\prime }}_{n}^{}/T_{P}^{},n=0,1,\cdots ,N^{\prime }-1$ represents the *n*-th hopping frequency point after sampling, and is expressed as

(16) $${f^{\prime }}_{n}\in f^{\prime }={\left[\underset{N\cdot N_{S}}{\underset{︸}{f_{0},f_{0},\cdots ,f_{0}}},\underset{N\cdot N_{S}}{\underset{︸}{f_{1},f_{1},\cdots ,f_{1}}},\cdots ,\underset{N\cdot N_{S}}{\underset{︸}{f_{M-1},f_{M-1},\cdots ,f_{M-1}}}\right]}_{}^{T}$$

$c^{\prime }\left(n\right)$ in (15) is the sampled phase-coding sequence given by

(17) $$c^{\prime }\left(n\right)\in c^{\prime }={\left[\underset{N_{S}}{\underset{︸}{c_{0}^{}\left(0\right),\cdots }}\underset{N_{S}}{\underset{︸}{,c_{0}^{}\left(1\right),\cdots }},\cdots ,\underset{N_{S}}{\underset{︸}{c_{0}^{}\left(N-1\right),\cdots }},\cdots ,\underset{N_{S}}{\underset{︸}{c_{m}^{}\left(n\right),\cdots }},\cdots ,\underset{N_{S}}{\underset{︸}{c_{M-1}^{}\left(N-1\right),\cdots }}\right]}_{}^{T}$$

Finally, ${p^{\prime }}_{n}\left(t\right)$ in (15) represents the ideal rectangular pulse with width $T_{S}^{}$.

It follows that the autocorrelation function of the FSK-PSK signal is given by

(18) $$\begin{array}{cc} \chi \left(\tau \right) & =\int_{-\infty }^{\infty }\sum_{n=0}^{N^{\prime }-1}c^{\prime }\left(n\right){p^{\prime }}_{n}\left(t\right)e^{j2\pi {f^{\prime }}_{n}t}\sum_{m=0}^{N^{\prime }-1}{c^{\prime }}_{}^{*}\left(m\right){p^{\prime }}_{m}\left(t+\tau \right)e^{-j2\pi {f^{\prime }}_{m}\left(t+\tau \right)}dt\\ & =\sum_{n=0}^{N^{\prime }-1}\sum_{m=0}^{N^{\prime }-1}c^{\prime }\left(n\right){c^{\prime }}_{}^{*}\left(m\right)e^{-j2\pi {f^{\prime }}_{m}\tau }\int_{-\infty }^{\infty }{p^{\prime }}_{n}\left(t\right){p^{\prime }}_{m}\left(t+\tau \right)e^{j2\pi \left({f^{\prime }}_{n}-{f^{\prime }}_{m}\right)t}dt \end{array}$$

Define $\begin{array}{cc} \tau =kT_{S}, & -(N^{\prime }-1)\leq k\leq N^{\prime }-1 \end{array}$. With some manipulation, (18) can be recast as

(19) $$\begin{array}{cc} \chi \left(kT_{S}^{}\right) & =\sum_{n=0}^{N^{\prime }-1}\sum_{m=0}^{N^{\prime }-1}c^{\prime }\left(n\right)c_{}^{\prime }*\left(m\right)e^{-j2\pi {f^{\prime }}_{m}kT_{S}^{}}\int_{-\infty }^{\infty }{p^{\prime }}_{n}\left(t\right){p^{\prime }}_{m}\left(t+kT_{S}^{}\right)e^{j2\pi \left({f^{\prime }}_{n}-{f^{\prime }}_{m}\right)t}dt\\ & =\sum_{n=0}^{N^{\prime }-1}c^{\prime }\left(n\right)c_{}^{\prime }*\left(n+k\right)e^{-j2\pi {f^{\prime }}_{n+k}kT_{S}^{}}\int_{-\infty }^{\infty }{p^{\prime }}_{n}\left(t\right){p^{\prime }}_{n+k}\left(t+kT_{S}^{}\right)e^{j2\pi \left({f^{\prime }}_{n}-{f^{\prime }}_{n+k}\right)t}dt\\ & =\sum_{n=0}^{N^{\prime }-1}c^{\prime }\left(n\right)c_{}^{\prime }*\left(n+k\right)e^{-j2\pi {f^{\prime }}_{n+k}kT_{S}^{}}\sin c\left[\left({f^{\prime }}_{n}-{f^{\prime }}_{n+k}\right)T_{S}\right]e^{j\pi \left({f^{\prime }}_{n}-{f^{\prime }}_{n+k}\right)\left(2n+1\right)T_{S}}\\ & =\sum_{n=0}^{N^{\prime }-1}c^{\prime }\left(n\right)c_{}^{\prime }*\left(n+k\right)\sin c\left(\frac{{\sigma^{\prime }}_{n}-{\sigma^{\prime }}_{n+k}}{N_{S}}\right)e^{j\pi \left(2n+1\right)\frac{{\sigma^{\prime }}_{n}-{\sigma^{\prime }}_{n+k}}{N_{S}}-j2\pi k\frac{{\sigma^{\prime }}_{n+k}}{N_{S}}} \end{array}$$

Since *T_S_* is a constant, $\chi \left(kT_{S}^{}\right)$ is written as $\chi \left(k\right)$ in the following text for simplicity. We further define

(20) $$H_{k}^{}=J_{k}\cdot D_{k}$$

where $J_{k}$ is the shift matrix, and

(21) $$D_{k}=\mathrm{diag}\left[\sin c\left(\frac{{\sigma^{\prime }}_{n}-{\sigma^{\prime }}_{n+k}}{N_{S}}\right)e^{j\pi \left(2n+1\right)\frac{{\sigma^{\prime }}_{n}-{\sigma^{\prime }}_{n+k}}{N_{S}}-j2\pi k\frac{{\sigma^{\prime }}_{n+k}}{N_{S}}}\left(n=0,1,\cdots ,N^{\prime }-1-k\right),0_{1\times k}\right]$$

Finally, the compact form of the autocorrelation function of the FSK-PSK signal is obtained as

(22) $$\begin{array}{cc} \chi \left(k\right)={c^{\prime }}_{}^{H}H_{k}c^{\prime }, & -(N^{\prime }-1)\leq k\leq N^{\prime }-1 \end{array}$$

## 3. Low Sidelobe Waveform Optimization Algorithm Based on ADMM (LSW-ADMM)

### 3.1. ADMM Algorithm

The ADMM algorithm is an effective way to solve separable convex optimization problems with multiple variables. By decomposing the original optimization problem into multiple easier-to-solve sub-problems and alternately solving them, the scale of the computational complexity can be reduced dramatically [35]. The effectiveness of this method in simplifying the complicated problem of radar waveform optimization has been demonstrated in [36,37,38,39].

The general ADMM framework is expressed as

(23) $$\begin{array}{cc} \min & f(x)+g(z)\\ s.t. & Ax+Bz=c \end{array}$$

Equation (23) can be written as an augmented Lagrange function as

(24) $$\begin{array}{c} L_{\rho }(x,z,\lambda )=f(x)+g(z)+\lambda^{T}(Ax+Bz-c)+\\ \frac{\rho }{2}{\left\|Ax+Bz-c\right\|}^{2} \end{array}$$

where ρ is the coefficient for the penalty term. The updating equations for the ADMM algorithm are given by

(25) $$\begin{array}{l} x^{\left(t+1\right)}=\underset{x}{\mathrm{argmin}}\;L_{\rho }(x,z^{\left(t\right)},\lambda^{\left(t\right)})\\ z^{\left(t+1\right)}=\underset{z}{\mathrm{argmin}}\;L_{\rho }(x^{\left(t+1\right)},z,\lambda^{\left(t\right)})\\ \lambda^{\left(t+1\right)}=\lambda^{\left(t\right)}+\rho (Ax^{\left(t+1\right)}+Bz^{\left(t+1\right)}-c) \end{array}$$

We set a small positive number δ as the convergence threshold, i.e., the algorithm converges for $\left\|x^{\left(t+1\right)}-z^{\left(t+1\right)}\right\|<\delta$.

### 3.2. LSW-ADMM Optimization Method

In this subsection, the phase-coding sequence is optimized to minimize the AF sidelobes of the FSK-PSK signal. This integrated sidelobe level (ISL) is defined as

(26) $$\mathrm{ISL}=\sum_{k\in \Psi }{c^{\prime }}_{}^{H}H_{k}c^{\prime }$$

where $\Psi =\left[k\left|N_{S}^{}\leq \left|k\right|\leq N^{\prime }\right.-1\right]$. To minimize the ISL in (26), the ADMM and the quasi-Newton method are employed to solve the convex optimization problem. The optimization problem is expressed as

(27) $$\begin{array}{ll} \underset{c}{\min} & \sum_{k\in \Psi }{c^{\prime }}_{}^{H}H_{k}c^{\prime }\\ s.t. & c\left(n\right)=e^{j2\pi k\left(n\right)/K}\;\;\;n=0,1,\cdots ,N-1;k\left(n\right)\in \left[0,1,\cdot \cdot \cdot K-1\right] \end{array}$$

Introducing an auxiliary variable c=z to the constraint, we can obtain

(28) $$\begin{array}{ccc} \underset{c}{\min} & \sum_{k\in \Psi }{c^{\prime }}_{}^{H}H_{k}c^{\prime }\\ s.t. & c=z\\ & z\left(n\right)=e^{j2\pi k\left(n\right)/K} & n=0,1,\cdots ,N-1;k\left(n\right)\in \left[0,1,\cdot \cdot \cdot K-1\right] \end{array}$$

The augmented Lagrange equation corresponding to (28) is expressed as

(29) Lρ(c,z,λ)=∑k∈Ψc′HHkc′+λrTRe(c−z)+λiTIm(c−z)+ρ2c−z2

where $\lambda_{r}$, $\lambda_{i}$ and ρ are Lagrange coefficients.

Define $u=(\lambda_{r}+j\lambda_{i})/\rho$, (29) can be recast as

(30) $$L_{\rho }(c,z,u)=\sum_{k\in \Psi }{c^{\prime }}_{}^{H}H_{k}c^{\prime }+\frac{\rho }{2}{\left\|c-z+u\right\|}^{2}-\frac{\rho }{2}{\left\|u\right\|}_{}^{2}$$

Define $c^{\left(t\right)}$,$z^{\left(t\right)},u^{\left(t\right)}$ as the values of c, z and u after *t* iterations. Given the initial value $c^{\left(0\right)},z^{\left(0\right)},u^{\left(0\right)}$, then the solution of the problem can be carried out according to the following steps

(1) Given $z^{\left(t\right)},u^{\left(t\right)}$, update $c^{\left(t+1\right)}$ as

(31) $$c^{\left(t+1\right)}=\mathrm{argmin}\left\{\sum_{k\in \Psi }{c^{\prime }}_{}^{H}H_{k}c^{\prime }+\frac{\rho }{2}{\left\|c-z^{\left(t\right)}+u^{\left(t\right)}\right\|}^{2}\right\}$$

This problem is an unconstrained optimization problem, which can be solved by the quasi-Newton method [40]. First, we define

c˜=Re(c′T)Im(c′T)T,A=Re(H)−Im(H)Im(H)Re(H), B=Im(H)Re(H)−Re(H)Im(H).

Then ${c^{\prime }}_{}^{H}H_{k}c^{\prime }$ is expressed as

(32) $${c^{\prime }}_{}^{H}H_{k}c^{\prime }=\sqrt{{\left({\tilde{c}}_{}^{T}A\left(k\right){\tilde{c}}_{}\right)}^{2}+{\left({\tilde{c}}_{}^{T}B\left(k\right)\tilde{c}\right)}^{2}}$$

(2) Given $c^{\left(t+1\right)},u^{\left(t\right)}$, update z as

(33) $$\begin{array}{cc} z^{\left(t+1\right)} & =\underset{z}{\mathrm{argmin}}\;{\left\|c^{\left(t+1\right)}-z+u^{\left(t\right)}\right\|}^{2}\\ s.t. & z\left(n\right)=e^{j2\pi k\left(n\right)/K}\;\;\;\;\;n=0,1,\cdots ,N-1;k\left(n\right)\in \left[0,1,\cdot \cdot \cdot K-1\right] \end{array}$$

Equation (33) could be recast as

(34) zt+1=argmaxz Re(ct+1+ut)Hzs.t.zn=ej2πkn/K,      n=0,1,⋯,N−1;kn∈0,1,⋅⋅⋅K−1

Since (34) is a linear equation and $z\left(n\right)$, n=0,1,⋯,N−1 are independent of each other, (34) can be decomposed into $k\left(n\right)$ sub-problems as

(35) zt+1n=argmaxkn Rect+1n+utn*⋅ztns.t.zn=ej2πkn/K,      kn∈0,1,⋅⋅⋅K−1

With some manipulation, the objective function in (35) could be written as

(36) Rect+1n+utn*⋅ztn=Rect+1n+utn⋅Reztn+Imct+1n+utn⋅Imztn=ct+1n+utn⋅ztn⋅cosct+1n+utn,ztn

Ignoring the constant coefficient, Equation (36) is simplified as

(37) $$\begin{array}{cc} z_{}^{\left(t+1\right)}\left(n\right) & \underset{k\left(n\right)}{=\mathrm{argmax}}\cos\langle c_{}^{\left(t+1\right)}\left(n\right)+u_{}^{\left(t\right)}\left(n\right),z_{}^{\left(t\right)}\left(n\right)\rangle \\ s.t. & z\left(n\right)=e^{j2\pi k\left(n\right)/K},\;\;\;\;\;\;k\left(n\right)\in \left[0,1,\cdot \cdot \cdot K-1\right] \end{array}$$

To minimize the angle between the vectors $z\left(n\right)=e^{j2\pi k\left(n\right)/K}$ and $c_{}^{\left(t+1\right)}\left(n\right)+u_{}^{\left(t\right)}\left(n\right)$ in the complex plane, the integer $k\left(n\right)(0\leq k\left(n\right)<K)$ is obtained as

(38) $$k\left(n\right)=\left\lfloor \frac{\arg\left[c_{}^{\left(t+1\right)}\left(n\right)+u_{}^{\left(t\right)}\left(n\right)\right]}{2\pi /K}+\frac{1}{2}\right\rfloor$$

where $\left\lfloor \cdot \right\rfloor$ stands for the floor function. The solution to (34) is obtained as

(39) $$z^{\left(t+1\right)}=\exp\left(j2\pi \frac{\left\lfloor K\cdot \arg\left(c^{\left(t+1\right)}+u^{\left(t\right)}\right)/2\pi +1/2\right\rfloor }{K}\right)$$

(3) Given $u^{\left(t\right)},\;c^{\left(t+1\right)},\;z^{\left(t+1\right)}$, update $u^{\left(t+1\right)}$ as

(40) $$u^{\left(t+1\right)}=u^{\left(t\right)}+c^{\left(t+1\right)}-z^{\left(t+1\right)}$$

The procedures of LSW-ADMM algorithm are as follows. To begin, initialize c,z, and u as $c^{\left(0\right)},z^{\left(0\right)},u^{\left(0\right)}$. Next, repeat step 1) to step 3) until the number of iterations reaches the upper limit—i.e., $\left|{\mathrm{ISL}}^{\left(t+1\right)}-{\mathrm{ISL}}^{\left(t\right)}\right|\leq \varepsilon$—with ε being the convergence threshold (or until the iteration stop condition is satisfied). The implementation steps of the LSW–ADMM algorithm are given in Table 1.

## 4. Anti-Slice-Repeater-Jamming Algorithm Based on ADMM (ASRJ-ADMM)

### 4.1. Intra-Pulse Slice Repeater Model

**Figure 2:**
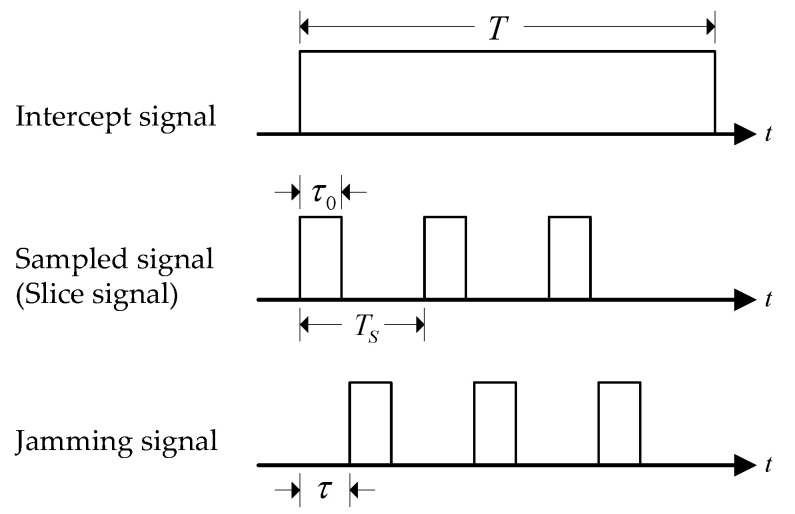
Schematic diagram of slice repeater jamming model.

The signal structure of the intra-pulse slice repeater jamming generated by the enemy jammer is shown in Figure 2. The jammer intercepts part of the radar signal in the time domain according to the sampling period to generate the intercepted signal, which can be regarded as multiplying the radar signal with an ideal rectangular pulse with a sampling period of $T_{s}$ and a width of $\tau_{0}$ to obtain an intra-pulse slice signal. The slice signal is processed by power amplification and delayed-forwarding to generate an intra-pulse slice repeater jamming signal. After the radar receives the intra-pulse slice repeater jamming, multiple deception targets are formed due to the coherence of the repeater jamming.

For the specific slice repeater jamming whose slice signal width is equal to the width of the FSK-PSK intra-sub-pulse signal, the intra-sub-pulse orthogonal waveform can be used to suppress the repeater jamming [41,42,43].

### 4.2. Intra-Pulse Slice Repeater Jamming Suppression Model

Suppose $N_{s}$ points are sampled with a sampling period of $T_{s}=T_{p}/N_{s}$, and the total number of sampling points is $N^{\prime }=N\cdot N_{s}$ for a FSK-PSK signal made of a single sub-pulse. Equation (2) is rewritten as

(41) $$\begin{array}{cc} u_{m}\left(t\right)=e^{j2\pi f_{m}^{}t}\sum_{n=0}^{N^{\prime }-1}{c^{\prime }}_{m}\left(n\right){p^{\prime }}_{n}\left(t\right), & 0\leq t\leq T_{c}^{} \end{array}$$

where ${p^{\prime }}_{n}\left(t\right)$ represents an ideal rectangular pulse with a width of $T_{S}$, and ${c^{\prime }}_{m}$ represents the phase-coding sequence of the *m*-th sampled sub-pulse signal, which is given by

(42) $${c^{\prime }}_{m}=\left[\underset{N_{S}}{\underset{︸}{c_{m}\left(0\right),\cdots ,c_{m}\left(0\right)}},\underset{N_{S}}{\underset{︸}{c_{m}\left(1\right)\cdots ,c_{m}\left(1\right)}},\cdots ,\underset{N_{S}}{\underset{︸}{c_{m}\left(N-1\right),\cdots ,c_{m}\left(N-1\right)}}\right]$$

According to the definition of the ambiguity function, it follows from (41) that

(43) $$\begin{array}{c} \chi_{ml}\left(\tau \right)\\ =\int_{-\infty }^{\infty }e^{j2\pi f_{m}t}\sum_{n=0}^{N^{\prime }-1}{c^{\prime }}_{m}\left(n\right){p^{\prime }}_{n}\left(t\right)\cdot e^{-j2\pi f_{l}\left(t+\tau \right)}\sum_{r=0}^{N^{\prime }-1}{c^{\prime }}_{l}^{*}\left(r\right){p^{\prime }}_{r}\left(t+\tau \right)dt\\ =e^{-j2\pi f_{l}\tau }\sum_{n=0}^{N^{\prime }-1}\sum_{r=0}^{N^{\prime }-1}{c^{\prime }}_{m}\left(n\right){c^{\prime }}_{l}^{*}\left(r\right)\int_{-\infty }^{\infty }{p^{\prime }}_{n}\left(t\right){p^{\prime }}_{r}\left(t+\tau \right)e^{j2\pi \left(f_{m}-f_{l}\right)t}dt \end{array}$$

By setting $\begin{array}{cc} \tau =kT_{S}, & -(N^{\prime }-1)\leq k\leq N^{\prime }-1 \end{array}$, (43) can be recast as

(44) $$\begin{array}{c} \chi_{ml}\left(kT_{S}\right)\\ =e^{j\pi \frac{\sigma_{m}^{}-\left(2k+1\right)\sigma_{l}^{}}{N_{S}^{}}}\sin c\left(\frac{\sigma_{m}^{}-\sigma_{l}^{}}{N_{S}^{}}\right)\sum_{n=0}^{N^{\prime }-1}{c^{\prime }}_{m}\left(n\right){c^{\prime }}_{l}^{*}\left(n+k\right)e^{j2n\pi \frac{\sigma_{m}^{}-\sigma_{l}^{}}{N_{S}^{}}} \end{array}$$

Since *T_p_* is a constant, $\chi_{ml}\left(kT_{p}\right)$ is simplified as $\chi_{ml}\left(k\right)$ in the following text. We further define

(45) $$H_{ml,k}=\xi_{ml,k}\cdot J_{k}^{}\cdot D_{ml}^{}$$

(46) $$D_{ml}=\mathrm{diag}(e^{0},e^{j2\pi \frac{\sigma_{m}^{}-\sigma_{l}^{}}{N_{S}^{}}},\cdot \cdot \cdot ,e^{j2\left(N^{\prime }-1\right)\pi \frac{\sigma_{m}^{}-\sigma_{l}^{}}{N_{S}^{}}})$$

(47) $$\xi_{ml,k}=e^{j\pi \frac{\sigma_{m}^{}-\left(2k+1\right)\sigma_{l}^{}}{N_{S}^{}}}\sin c\left(\frac{\sigma_{m}^{}-\sigma_{l}^{}}{N_{S}^{}}\right)$$

The cross-correlation of the sub-pulses of the FSK-PSK signal is then obtained in the discrete form as

(48) $$\begin{array}{cc} \chi_{ml}\left(k\right)={c^{\prime }}_{l}^{H}H_{ml,k}{c^{\prime }}_{m}, & -(N^{\prime }-1)\leq k\leq N^{\prime }-1 \end{array}$$

when m=l, (48) represents the discrete-form of the AF of the sub-pulse signal

(49) $$\begin{array}{cc} \chi_{m}\left(k\right)={c^{\prime }}_{m}^{H}H_{m,k}{c^{\prime }}_{m}, & -(N^{\prime }-1)\leq k\leq N^{\prime }-1 \end{array}$$

where $H_{m,k}=e^{-2j\pi k\frac{\sigma_{m}^{}}{N_{S}^{}}}\cdot J_{k}^{}$.

To ensure the near-orthogonality between the sub-pulses of the FSK-PSK signal, optimization problem can be formulated to minimize the AF sidelobes and the weighted sum of the cross-correlations between the sub-pulses. Assume that the frequency-hopping coding sequence is fixed, we seek to optimize the discrete phase-coding sequence. The objective function of the optimization problem is formulated as

(50) $$\delta =\alpha {\sum_{m}\sum_{k\in \Psi }\left|\chi_{m}(k)\right|}^{2}+(1-\alpha ){\sum_{m\neq l}\sum_{k\in \Phi }\left|\chi_{ml}(k)\right|}^{2}$$

where $\Psi =\left[k|N_{s}\leq \left|k\right|\leq N^{\prime }-1\right]$, $\Phi =\left[k|0\leq \left|k\right|\leq N^{\prime }-1\right]$, and $\alpha \in \left[0,1\right]$ is the weighting coefficient. It could be easily observed from (50) that when α=1, the sole purpose of (50) is to minimize the AF sidelobes. While for α=0, the sole purpose of (50) switches to the minimization of the cross-correlations.

### 4.3. ASRJ-ADMM Optimization Method

The ADMM architecture and the quasi-Newton method are used to minimize the weighted sum of the AF sidelobes and the cross-correlations of the sub-pulses of the FSK-PSK signal. The optimization problem is formulated as

(51) $$\begin{array}{l} \begin{array}{cc} \underset{c}{\min} & \alpha \sum_{m}\sum_{k\in \Psi }{\left|\chi_{m}\left(k\right)\right|}^{2}+\left(1-\alpha \right)\sum_{m\neq l}\sum_{k\in \Phi }{\left|\chi_{ml}\left(k\right)\right|}^{2}\\ s.t. & \begin{array}{l} \begin{array}{cc} c_{m}\left(n\right)=e^{j2\pi k_{m}^{}\left(n\right)/K} & n=0,1,\cdots ,N-1; \end{array}\\ m=0,1,\cdots ,M-1;k_{m}^{}\left(n\right)\in \left[0,1,\cdot \cdot \cdot K-1\right] \end{array} \end{array} \end{array}$$

Equation (51) can be recast as

(52) $$\begin{array}{l} \begin{array}{cc} \underset{c}{\min} & \alpha \sum_{m}\sum_{k\in \Psi }{\left|{c^{\prime }}_{m}^{H}H_{m,k}{c^{\prime }}_{m}\right|}^{2}+\left(1-\alpha \right)\sum_{m\neq l}\sum_{k\in \Phi }{\left|{c^{\prime }}_{l}^{H}H_{ml,k}{c^{\prime }}_{m}\right|}^{2}\\ s.t. & \begin{array}{l} \begin{array}{cc} c_{m}\left(n\right)=e^{j2\pi k_{m}^{}\left(n\right)/K} & n=0,1,\cdots ,N-1; \end{array}\\ m=0,1,\cdots ,M-1;k_{m}^{}\left(n\right)\in \left[0,1,\cdot \cdot \cdot K-1\right] \end{array} \end{array} \end{array}$$

Introducing an auxiliary variable c=z to the constraint, we can obtain

(53) $$\begin{array}{cc} \underset{c}{\min} & \alpha \sum_{m}\sum_{k\in \Psi }{\left|{c^{\prime }}_{m}^{H}H_{m,k}{c^{\prime }}_{m}\right|}^{2}+\left(1-\alpha \right)\sum_{m\neq l}\sum_{k\in \Phi }{\left|{c^{\prime }}_{l}^{H}H_{ml,k}{c^{\prime }}_{m}\right|}^{2}\\ s.t. & c=z\\ & \begin{array}{l} z_{m}\left(n\right)=e^{j2\pi k_{m}^{}\left(n\right)/K},n=0,1,\cdots ,N-1;\\ m=0,1,\cdots ,M-1;k_{m}^{}\left(n\right)\in \left[0,1,\cdot \cdot \cdot K-1\right] \end{array} \end{array}$$

Note that the problem in (52) is equivalent to that in (53). The augmented Lagrange equation corresponding to (53) is expressed as

(54) Lρ(c,z,λ)=α∑m∑k∈Ψc′mHHm,kc′m2+1−α∑m≠l∑k∈Φc′lHHml,kc′m2+λrTRe(c−z)+λiTIm(c−z)+ρ2c−z2

where $\lambda_{r}$, $\lambda_{i}$, and ρ are Lagrange coefficients.

Let $u=(\lambda_{r}+j\lambda_{i})/\rho$, then (54) can be written as

(55) $$\begin{array}{cc} L_{\rho }\left(c,z,u\right)= & \alpha \sum_{m}\sum_{k\in \Psi }{\left|{c^{\prime }}_{m}^{H}H_{m,k}{c^{\prime }}_{m}\right|}^{2}+\left(1-\alpha \right)\sum_{m\neq l}\sum_{k\in \Phi }{\left|{c^{\prime }}_{l}^{H}H_{ml,k}{c^{\prime }}_{m}\right|}^{2}+\\ & \frac{\rho }{2}{\left\|c-z+u\right\|}^{2}-\frac{\rho }{2}{\left\|u\right\|}^{2} \end{array}$$

The implementation procedures of the ASRJ-ADMM algorithm are summarized in Table 2. To begin, initialize c,z, and u as $c^{\left(0\right)},z^{\left(0\right)},u^{\left(0\right)}$. Next, repeat steps b) to e) until the number of iterations reaches the upper limit—i.e., $\left|\delta^{\left(t+1\right)}-\delta^{\left(t\right)}\right|\leq \varepsilon$—with ε being a small positive number (or until the iteration stop condition is satisfied).

## 5. Computational Complexity Analysis

In the ADMM algorithm, (31) and (53) dominate the main computation. We need to calculate the gradient of (31) and (53). Considering the subpulse length $N^{\prime }$, we can suppose Ψ contains $\alpha {N^{\prime }}^{2}$ points, where 0 < α < 1. Then, the computation of gradient contains

(56) $$\alpha \cdot {N^{\prime }}^{2}\cdot 2\cdot (N_{\mathrm{DT}}\cdot 8\cdot {N^{\prime }}^{3}+N_{\mathrm{DT}}\cdot 4\cdot {N^{\prime }}^{2})+2N_{\mathrm{DT}}N^{\prime }$$

times of real number multiplications, and

(57) $$\alpha \cdot {N^{\prime }}^{2}\cdot 2\cdot (N_{\mathrm{DT}}\cdot 8\cdot {N^{\prime }}^{3}+N_{\mathrm{DT}}\cdot 4\cdot {N^{\prime }}^{2})+4N_{\mathrm{DT}}N^{\prime }$$

times of real number additions. Therefore, the computational complexity of ADMM algorithm is on the order of $o(N_{\mathrm{DT}}{N^{\prime }}^{5})$.

We note that GA algorithm can also be successfully applied in discrete-phase sequence design problems. However, with increased variables in FSK-PSK optimization problem, the efficiency of GA algorithm is not satisfactory.

In each iteration of GA algorithm, the number of variables in c is $N^{\prime }$, and for each variable $c_{n}$, we need to calculate the number $\alpha {N^{\prime }}^{2}$ locations in the DAF. The problem of $c_{n}$ in [31], has the computational complexity of $o(N_{\mathrm{DT}}{N^{\prime }}^{3})$.The total computational complexity of GA algorithm is $o(N_{\mathrm{DT}}{N^{\prime }}^{6})$. Therefore, the computational complexity of GA algorithm is higher than that of the ADMM algorithm.

## 6. Numerical Experiments

In this section, we provide two simulation examples to demonstrate the performance of the two optimization algorithms proposed in Section 3 and Section 4. The first example shows that the LSW-ADMM algorithm proposed in this paper has faster convergence speed and lower computational complexity compared with the GA algorithm. Moreover, the FSK-PSK signal optimized with the LSW-ADMM algorithm exhibits lower AF sidelobes, which in turn contributes to better detection performance. The second example shows that, compared with the conventional FSK-PSK signal modulated with the classic coding sequence, the FSK-PSK signal optimized with the ASRJ-ADMM algorithm possesses better anti-jamming capability due to a higher-order of randomness of the radar signal. Moreover, unlike the GA algorithm, which suppresses the AF sidelobes at the cost of higher cross-correlations, the ASRJ-ADMM algorithm can realize joint optimization of the AF and the cross-correlation. The FSK-PSK signal optimized with the ASRJ-ADMM algorithm guarantees near-orthogonality between sub-pulses, hence has the potential to suppress the repeater jamming of specific intra-pulse slices effectively.

**Figure 3:**
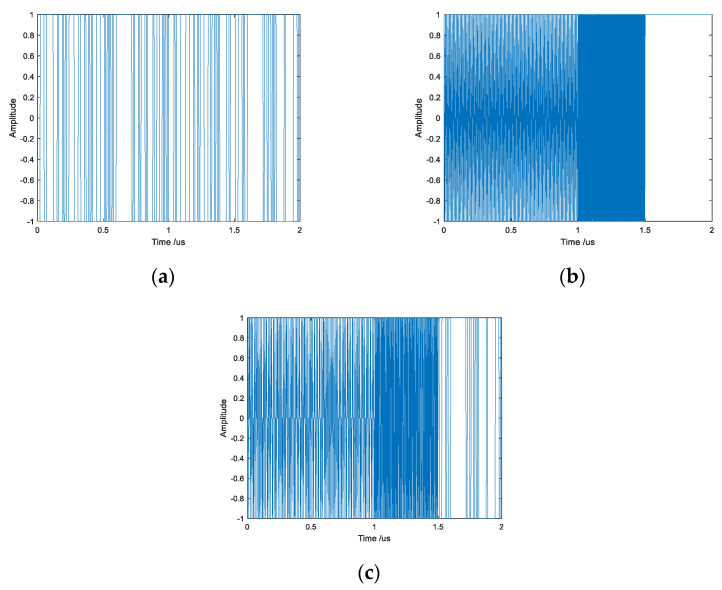
PSK, FSK, and Hybrid FSK-PSK signals. (**a**) PSK signal. (**b**) FSK signal. (**c**) FSK-PSK signal.

**Figure 4:**
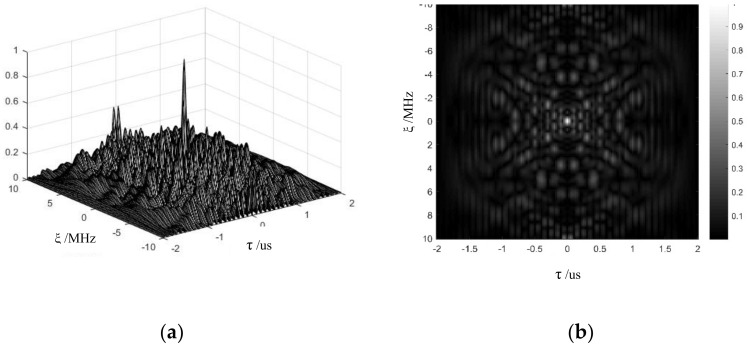
FSK-PSK signal ambiguity function. (**a**) FSK-PSK signal ambiguity function. (**b**) FSK-PSK signal ambiguity diagram.

Assuming that T=2us, $T_{P}^{}=0.1us$, and $\sigma_{m}^{}=\left\{1,3,2,4\right\}$, The phase encoding sequence is a random two-phase code. The PSK, FSK, and hybrid FSK-PSK are plotted in Figure 3. The FSK-PSK signal ambiguity function is shown in Figure 4. The FSK-PSK signal ambiguity function is a pushpin ambiguity function, and the FSK-PSK signal has no range-Doppler coupling problem.

**Figure 5:**
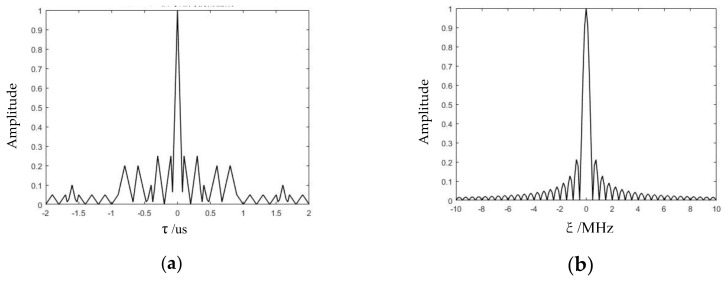
Zero-delay cut and the zero-Doppler cut of the 2D ambiguity function. (**a**) Zero-Doppler cut. (**b**) Zero-delay cut.

Let τ=0 and ξ=0, the zero-delay cut and the zero-Doppler cut of the 2D ambiguity function shown in Figure 5 could be obtained, which are depicted in Figure 5a,b, respectively. The velocity ambiguity function, main peak amplitude, and Doppler tolerance of FSK-PSK signals are all equivalent to PSK signals.

### 6.1. Optimization of the AF of the FSK-PSK Signal

According to the optimization model in (21), we define its average integrated sidelobe level (AISL) as

(58) $$\mathrm{AISL}=\frac{1}{2\left(N^{\prime }-N_{S}^{}\right)}\sum_{k\in \Psi }{c^{\prime }}_{}^{H}H_{k}c^{\prime }$$

where $\Psi =\left[k\left|N_{S}^{}\leq \left|k\right|\leq N^{\prime }\right.-1\right]$ represents the sidelobe range of the AF of the FSK-PSK signal.

**Figure 6:**
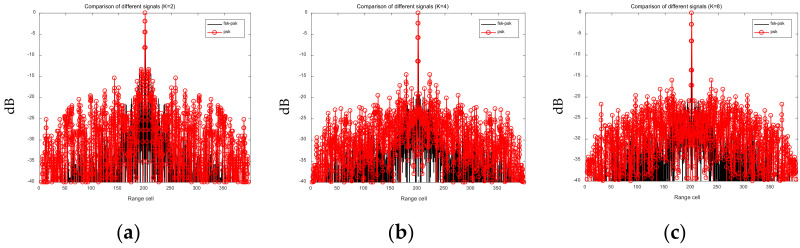
AISL comparison of the FSK-PSK signal and the PSK signal. (**a**) *K* = 2. (**b**) *K* = 4. (**c**) *K* = 8.

Assume that the number of sub-pulses is *M* = 4, the frequency hopping coefficient is $\sigma =\{\sigma_{m}^{},m=0,\cdots ,M-1\}=\{1,2,3,4\}$, the sub-pulse signal phase-coding length is N=50, and the discrete phase number is K=2/4/8. We also assume that the phase-coding sequence of the PSK signal is identical to that of the FSK-PSK signal. The AISL of the FSK-PSK signal and the PSK signal are plotted in in Figure 6. It can be seen from Figure 6 that the AISL of the FSK-PSK signal is significantly lower than that of the PSK signal. The results are summarized in Table 3.

**Figure 7:**
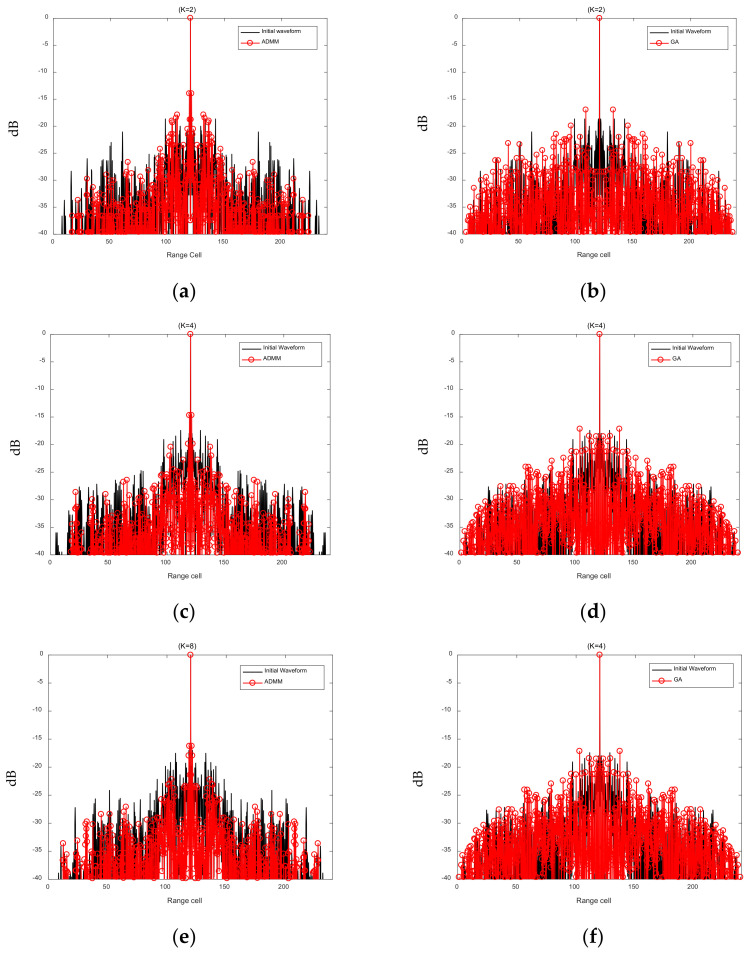
Comparison of FSK-PSK signal AF before and after optimization. (**a**) LSW-ADMM optimization (*K* = 2). (**b**) GA optimization (*K* = 2). (**c**) LSW-ADMM optimization (*K* = 4). (**d**) GA optimization (*K* = 4). (**e**) LSW-ADMM optimization (*K* = 8). (**f**) GA optimization (*K* = 8).

Next, we set the maximum number of the iterations of the optimization algorithm to 50, then use the LSW-ADMM and the GA algorithm to optimize the same initial FSK-PSK signal. The AFs before and after optimization when *K* takes on different values (*K* = 2, *K* = 4, *K* = 8) are shown in Figure 7. The black lines represent the initial waveforms before optimization, the red lines in Figure 7a,c,e represents the AFs of the FSK-PSK signals optimized with the LSW-ADDM, and the red lines in Figure 7b,d,f represents the AFs of the FSK-PSK signals optimized with the GA.

It can be seen clearly from Figure 6 that although the AF sidelobes of the FSK-PSK signal become lower by applying both optimization algorithms, the LSW-ADMM algorithm yields much lower AF side-lobes than that of the GA algorithm. Table 4 summarizes the AISL before and after waveform optimization with the LSW-ADMM algorithm and the GA algorithm. As can be seen from Table 4, the LSW-ADMM algorithm offers an AISL 3–5 dB lower than that of the GA algorithm. Specifically, with the increase of *K* from 2 to 8, the advantage of the LSW-ADMM algorithm becomes more obvious. However, as the number of discrete phases *K* further increases, the optimization effect of LSW-ADMM algorithm may not improve accordingly due to the limitation set by the coefficients for the penalty term in the Lagrange expression (see Section 3.2, though a detailed analysis of this phenomenon is beyond the scope of this work).

**Figure 8:**
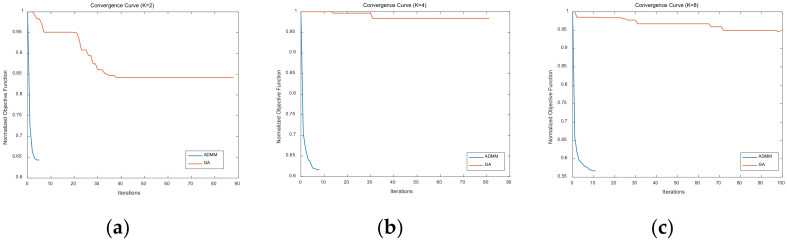
Convergence speed of the LSW-ADMM algorithm and the GA algorithm. (**a**) *K* = 2. (**b**) *K* = 4. (**c**) *K* = 8.

Figure 8 shows the convergence speed of the LSW-ADMM algorithm and the GA algorithm. It could be seen that the LSW-ADMM algorithm converges in less than 10 iterations, while the GA algorithm takes much more iterations to converge. As the number of discrete phase increases, the number of iterations of the LSW-ADMM algorithm requires to converge will slightly increase. Hence, we could conclude that the optimization effect of the GA algorithm is strictly constrained by the number of optimization iterations that are allowed within a specified period of time.

### 6.2. Joint Optimization of Intra-Pulse Sub-Pulse Correlation Function

In this example, we set the number of FSK-PSK signal sub-pulses as *M* = 4, the frequency hopping coefficient as $\sigma =\{\sigma_{m}^{},m=0,\cdots ,M-1\}=\{1,2,3,4\}$, the phase-coding length of the sub-pulse as N = 50, the number of discrete phases *K* = 2, the weighting coefficient of the optimization model as $\alpha =\left\{0,0.1,\cdots ,1\right\}$, and the upper limit of the optimization algorithm iteration number as 50. Identical initial waveforms are employed to test the waveform optimization performance of the ASRJ-ADMM algorithm and the GA algorithm. The auto-correlation average sidelobe level (AC-ASL) and cross-correlation average level (CC-AL) of the radar signals before and after optimization are shown in Figure 8. The AC-ASL and the CC-AL are defined, respectively, as

(59) $$\begin{array}{c} \mathrm{AC}-\mathrm{ASL}=\frac{1}{2\left(N^{\prime }-N_{S}^{}\right)\cdot M}\sum_{m}\sum_{k\in \Psi }{\left|\chi_{m}\left(k\right)\right|}^{2}\\ \mathrm{CC}-\mathrm{AL}=\frac{2}{\left(2N^{\prime }-1\right)\cdot M\left(M-1\right)}\sum_{m\neq l}\sum_{k\in \Phi }{\left|\chi_{ml}\left(k\right)\right|}^{2} \end{array}$$

where $\Psi =\left[k\left|N_{S}^{}\leq \left|k\right|\leq N^{\prime }\right.-1\right]$ and $\Phi =\left[k\left|0\leq \left|k\right|\leq N^{\prime }\right.-1\right]$.

**Figure 9:**
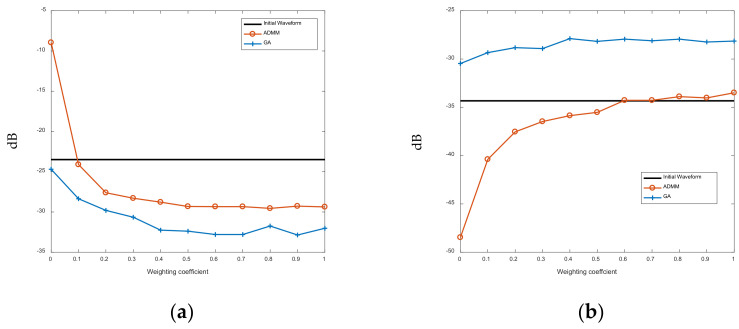
The AC-ASL and the CC-AL of the FSK-PSK signal before and after optimization. (**a**) AC-ASL result. (**b**) CC-AL result.

It can be seen from Figure 9a that as the weighting coefficient increases, the AC-ASL of the FSK-PSK signals optimized with the ASRJ-ADMM algorithm and the GA algorithm decreases gradually, while the AC-ASL of the FSK-PSK signal untouched by the waveform optimization algorithms remains constant. It could also be seen that the AC-ASL offered by the GA algorithm is slightly lower than that of the ASRJ-ADMM algorithm by 2–3 dB. However, this marginal performance gain of the GA algorithm over the ASRJ-ADMM algorithm is at the cost of higher CC-AL. As shown in Figure 9b, although the CC-AL of the proposed ASRJ-ADMM algorithm increases with the weighting coefficient, its maximum value is approximately equal to that of the FSK-PSK signal untouched by either optimization algorithms, and is 5–6 dB lower than that of the FSK-PSK signal optimized with the GA algorithm.

**Figure 10:**
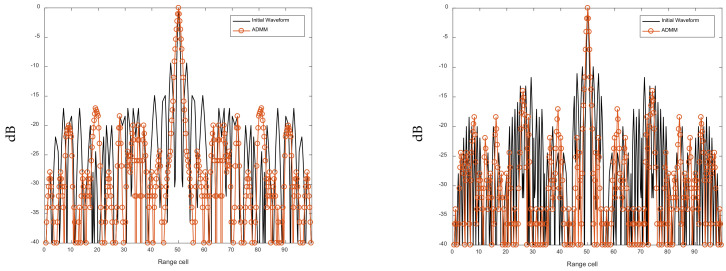

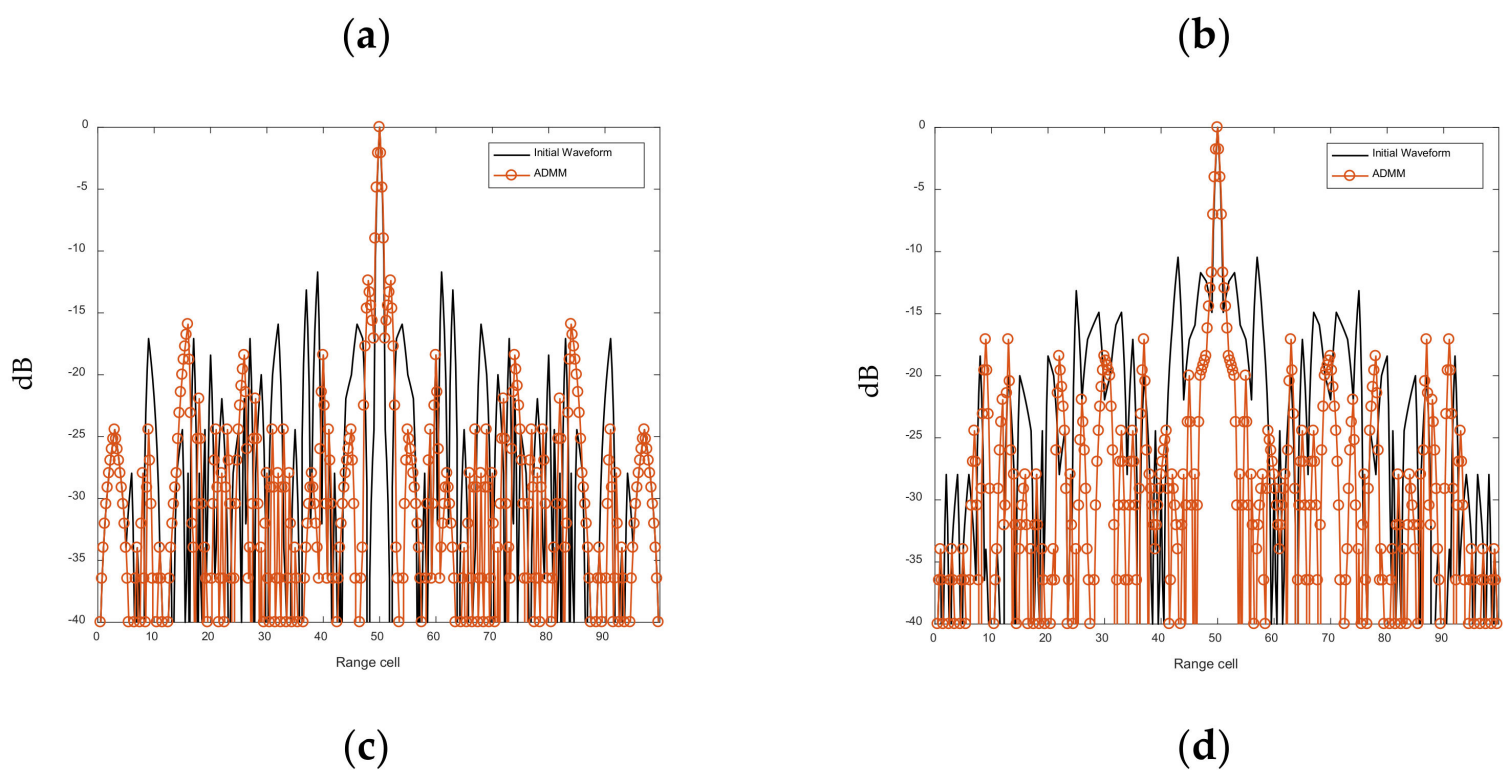
AFs of sub-pulse #1–4 of the FSK-PSK signal before and after optimization. (**a**) AF of Sub-pulse #1. (**b**) AF of Sub-pulse #2. (**c**) AF of Sub-pulse #3. (**d**) AF of Sub-pulse #4.

**Figure 11:**
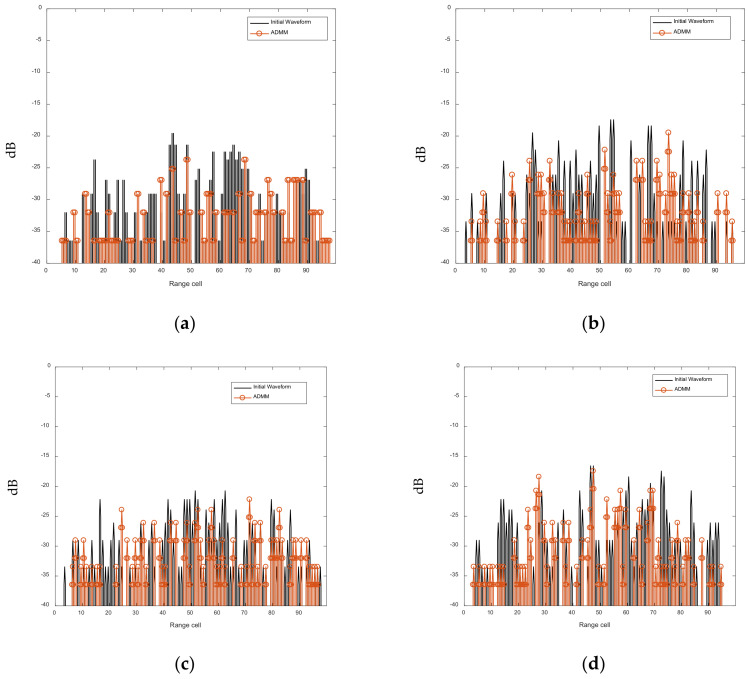

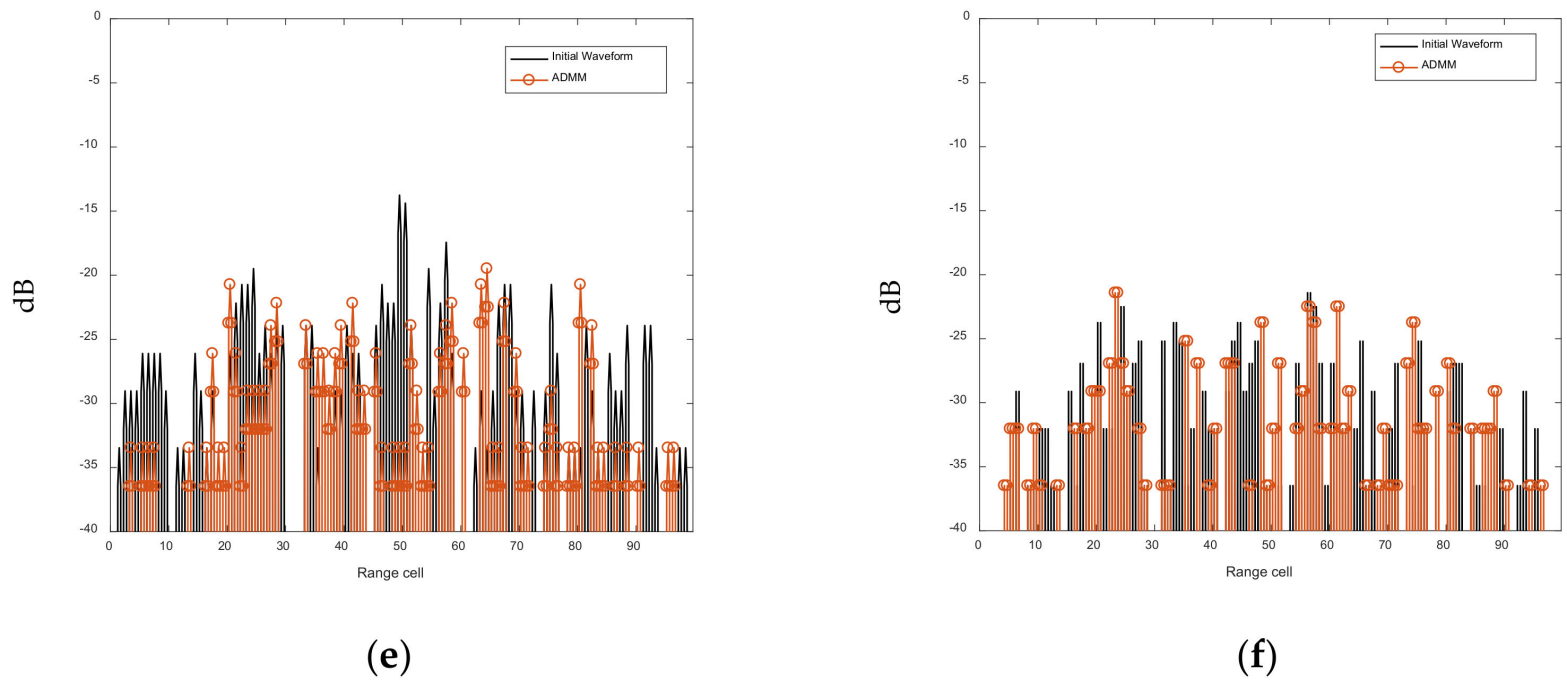
Cross-correlation of the subpulses of the FSK-PSK signals before and after optimization. (**a**) Cross-correlation of sub-pulse 1 and 2. (**b**) Cross-correlation of sub-pulse 1 and 3. (**c**) Cross-correlation of sub-pulse 1 and 4. (**d**) Cross-correlation of sub-pulse 2 and 3. (**e**) Cross-correlation of sub-pulse 2 and 4. (**f**) Cross-correlation of sub-pulse 3 and 4.

Figure 10 and Figure 11 show that, after the ADMM-based optimization process, the AF sidelobes and cross-correlations of the FSK-PSK signal are suppressed. Table 5 compares the AC-ASL and CC-AL before and after the ASRJ-ADMM optimization. It can be seen from Table 5 that both the AC-ASL and CC-AL are suppressed with the ASRJ-ADMM algorithm. However, based on the preliminary simulation results that are not presented in this work, as the number of discrete phases *K* further increases, the optimization effect may not improve accordingly.

By comparing the iteration convergence curve of the optimization algorithm in Figure 8 and the optimization algorithm time in Table 6, we can find that—compared with GA algorithm—LSW-ADMM and MSSD-ADMM algorithms have fewer iterations, faster convergence time, and better optimization effect.

## 7. Conclusions

In this work, two novel waveform optimization algorithms based on ADMM are proposed within the general FSK-PSK hybrid modulation framework for AF sidelobe suppression and waveform orthogonality enhancement. Specifically, the LSW-ADMM waveform optimization algorithm proposed in this work yields radar signals with AF sidelobes significantly lower than those of the conventional FSK-PSK signals. Meanwhile, the ASRJ-ADMM algorithm generates radar signals with outstanding anti-slice-repeater-jamming capability. In the follow-up works, we plan to further improve the detection performance and the anti-jamming capability of the FSK-PSK signal by jointly optimizing the frequency-hopping coding sequence and phase coding sequence.

## Figures and Tables

**Table 1:** LSW-ADMM steps.

LSW-ADMM
(a) Set t as the number of iterations. Initialize the algorithm with t=0 and set $c^{\left(0\right)},z^{\left(0\right)},u^{\left(0\right)};$
(b) $\mathrm{Using}\;\mathrm{quasi}\;\mathrm{Newton}\;\mathrm{method}\;\mathrm{to}\;\mathrm{solve}\;\mathrm{the}\;\mathrm{problem}c^{\left(t+1\right)}=\mathrm{argmin}\left\{\sum_{k\in \Psi }{c^{\prime }}_{}^{H}H_{k}c^{\prime }+\frac{\rho }{2}{\left\|c-z^{\left(t\right)}+u^{\left(t\right)}\right\|}^{2}\right\};$
(c) $z^{\left(t+1\right)}=\exp\left(j2\pi \frac{\left\lfloor K\cdot \arg\left(c^{\left(t+1\right)}+u^{\left(t\right)}\right)/2\pi +1/2\right\rfloor }{K}\right);$
(d) $u^{\left(t+1\right)}=u^{\left(t\right)}+c^{\left(t+1\right)}-z^{\left(t+1\right)};$
(e) t←t+1;
(f) If the convergence condition is satisfied, the algorithm is completed; otherwise, returns to (b)

**Table 2:** ASRJ-ADMM steps.

ASRJ-ADMM
(a) Set *t* as the number of iterations. Initialize the algorithm with t=0 and set $c^{\left(0\right)},z^{\left(0\right)},u^{\left(0\right)};$
(b) Using quasi-Newton method to solve the problem $\begin{array}{ll} c^{\left(t+1\right)}= & \underset{c}{\arg\;\min}\left\{\alpha \sum_{m}\sum_{k\in \Psi }{\left|{c^{\prime }}_{m}^{H}H_{m,k}{c^{\prime }}_{m}\right|}^{2}\right.\\ & \left.+\left(1-\alpha \right)\sum_{m\neq l}\sum_{k\in \Phi }{\left|{c^{\prime }}_{l}^{H}H_{ml,k}{c^{\prime }}_{m}\right|}^{2}+\frac{\rho }{2}{\left\|c-z^{\left(t\right)}+u^{\left(t\right)}\right\|}^{2}\right\}; \end{array}$
(c) $z^{\left(t+1\right)}=\exp\left(j2\pi \frac{\left\lfloor K\cdot \arg\left(c^{\left(t+1\right)}+u^{\left(t\right)}\right)/2\pi +1/2\right\rfloor }{K}\right);$
(d) $u^{\left(t+1\right)}=u^{\left(t\right)}+c^{\left(t+1\right)}-z^{\left(t+1\right)};$
(e) t←t+1;
(f) If the convergence condition is satisfied, the algorithm is completed. Otherwise, returns to (b)

**Table 3:** AISL comparison between FSK-PSK signal and PSK signal (unit: dB).

	*K* = 2	*K* = 4	*K* = 8
FSK-PSK signal	−36.3435	−35.4542	−35.2794
PSK signal	−28.8652	−29.2404	−29.0089

**Table 4:** AISL comparison before and after optimization (unit: dB).

	*K* = 2	*K* = 4	*K* = 8
Not optimized	−36.3435	−35.4542	−35.2794
LSW-ADMM	−39.8323	−39.2367	−40.2767
GA	−36.9693	−35.7298	−35.8229

**Table 5:** AC-ASL and CC-AL of the FSK-PSK signal before and after optimization (unit: dB).

Discrete Phase	*K* = 2 AC-ASL CC-AL	*K* = 4 AC-ASL CC-AL	*K* = 8 AC-ASL CC-AL
Not optimized	−23.4898 −34.3268	−23.5395 −33.2408	−23.0620 −32.9777
LSW-ADMM	−27.6087 −37.5398	−26.8966 −37.0582	−28.5616 −37.6952

**Table 6:** Comparison of optimization time between MSSD-ADMM and GA.

	K=2	K=4	K=8
LSW-ADMM	589 s	647 s	790 s
ASRJ-ADMM	623 s	765 s	810 s
GA	2305 s	2465 s	2725 s

## Data Availability

The datasets generated during the current study are not publicly available but are available from the corresponding author on reasonable request.
